# Allocation of TSPO-PET signals to cellular and extracellular compartments in dependence of blood-brain barrier disruption in experimental glioblastoma

**DOI:** 10.1007/s00259-026-08187-5

**Published:** 2026-09-24

**Authors:** Leonie Hoermann, Fatma Burcu Seker, Emanuel Joseph, Sabrina V Kirchleitner, Jens Blobner, Lea H Kunze, Justus F Thevis, Selina Hummel, Amelie L Englert, Karin Wind-Mark, Adrien Holzgreve, Simon Lindner, Rudolf A Werner, Louisa von Baumgarten, Nathalie L Albert, Nikolaus Plesnila, Matthias Brendel, Laura M Bartos

**Affiliations:** 1 Department of Nuclear Medicine, LMU University Hospital, LMU Medizin, Ludwig-Maximilians-Universität München, Munich, Germany; 2 Institute for Stroke and Dementia Research, LMU University Hospital, LMU Medizin, Ludwig-Maximilians-Universität München, Munich, Germany; 3 Munich Cluster for Systems Neurology (SyNergy), Munich, Germany; 4 German Center for Neurodegenerative Diseases (DZNE) Munich, Munich, Germany; 5 Department of Neurosurgery, LMU University Hospital, LMU Medizin, Ludwig-Maximilians-Universität München, Munich, Germany; 6 Russell H. Morgan Department of Radiology and Radiological Sciences, Johns Hopkins School of Medicine, Baltimore, MD USA; 7 Department of Neurology, LMU University Hospital, LMU Medizin, Ludwig-Maximilians-Universität München, Munich, Germany; 8 German Cancer Consortium (DKTK), Partner Site Munich, A Partnership Between the DKFZ Heidelberg and the University Hospital of the LMU, Munich, Germany; 9 Bavarian Cancer Research Center (BZKF), Partner Site Munich, Munich, Germany; 10 Institute of Clinical Neuroimmunology, LMU University Hospital, LMU Medizin, Ludwig-Maximilians-Universität München, Munich, Germany; 11 Biomedical Center (BMC), LMU Medizin, Ludwig-Maximilians-Universität München, Martinsried, Germany

**Keywords:** TSPO, Glioblastoma, Blocking, Specificity, Blood-brain barrier

## Abstract

**Background:**

Translocator protein (TSPO)-PET imaging facilitates monitoring of glioblastoma in preclinical models and patients. However, specificity of TSPO-PET signals remains to be investigated. In this study, we aimed to decipher exact contributions of cellular and extracellular compartments including the impact of blood-brain barrier (BBB) disruption to TSPO-PET signals in an experimental glioblastoma mouse model.

**Methods:**

Mice with implanted glioblastoma (SB28; *n* = 36 early-stage, *n* = 39 late-stage; GFP(+)) were injected with the TSPO tracer [^18^F]GE-180 (21 MBq ± 1.5 MBq). Dynamic 60 min microPET scans were performed with and without prior blocking by excessive cold radiotracer. Tumors were dissociated, followed by cell sorting of tumor cells, tumor-associated microglia/macrophages (TAMs, CD11b(+)) and non-tumor/non-TAM (i.e. remaining) cells with subsequent gamma emission measures and flow cytometry (scRadiotracing) to calculate radioactivity per single cell. PET signals and single-cell tracer uptake in tumors and non-lesional hemispheres were compared between blocked and unblocked conditions. *N* = 10 mice were intravenously injected with fluorescent Dextran to investigate BBB disruption by confocal microscopy in correlation with TSPO-PET signals of the same animals.

**Results:**

TSPO-PET indicated strong but incomplete signal reduction after blocking in tumors (early-stage: -72%; late-stage: -59%, *p* < 0.001) and contralateral hemispheres (early stage: -65%, late-stage: -63%, *p* < 0.001) compared to unblocked animals. We found nearly complete blocking of radiotracer uptake across all analyzed cell fractions (tumor cells: -95%, *p* = 0.0039; TAMs: -98%, *p* < 0.0001; remaining cells: -99%, *p* = 0.0033) compared to unblocked mice, regardless of tumor stages. There was a significant correlation of PET signal reduction (*R*=-0.824, *p* = 0.0034) as well as residual PET signal upon blocking (*R* = 0.809, *p* = 0.0046) with Dextran intensity.

**Conclusion:**

Specificity of TSPO-PET signals in experimental glioblastoma reaches 72% in early-stage tumors, but decreases to 59% in late-stage SB28 tumors, due to progressive contributions of BBB disruption. Non-specific TSPO tracer uptake is driven by BBB disruption and fully allocated to the extracellular compartment, whereas cellular TSPO tracer uptake shows strong specificity.

**Supplementary Information:**

The online version contains supplementary material available at https://doi.org/10.1007/s00259-026-08187-5.

## Background

Median survival of glioblastoma, the most common malignant primary brain tumor in adults, remains less than one year after diagnosis, warranting new therapy and monitoring strategies through innovative research [1]. Magnetic resonance imaging (MRI) is the primarily used imaging method in glioblastoma, although known to often underestimate tumor expansion [2]. As supramarginal resection increases progression-free survival [3], better knowledge of tumor margins and the tumor microenvironment (TME) could optimize surgery planning and therefore patient treatment. Positron emission tomography (PET) has hence become increasingly more important for assessment of the glioblastoma TME, with focus on new and improved radiotracers [4, 5]. [^18^F]GE-180 was developed as a ligand for the 18-kDa translocator protein (TSPO), located in outer mitochondrial membranes of activated myeloid cells within the TME, i.e. tumor associated microglia/macrophages (TAMs) [6]. However, TSPO is also expressed in glioma tumor cells [7, 8], which even constitute the main source of cellular TSPO-PET signal in glioblastoma [9]. Nonetheless, specificity of cellular signals, contribution of extracellular compartments and to which extent both components are dependent on blood-brain barrier (BBB) permeability remain to be investigated.

This has led to controversial discussion on using TSPO-PET, particularly [^18^F]GE-180, in glioblastoma [10, 11]. Therefore, we leveraged the tracer´s supposed BBB dependency and exploited our novel approach of cell sorting after radiotracer injection (scRadiotracing) [12] to pinpoint the impact of BBB disruption on TSPO-PET signals in glioblastoma. First, we used a syngeneic glioblastoma mouse model and conducted [^18^F]GE-180 PET scans with and without prior saturation of TSPO binding sites by non-radioactive GE-180 (i.e. blocking) to quantify the percentage of specific signal. Second, we performed scRadiotracing to investigate cellular specificity of the TSPO-PET signal in tumor cells, TAMs (CD11b(+)) and non-tumor/non-TAM (i.e. remaining) cells. Third, confocal microscopy upon intravenous administration of Dextran [13] was used to quantify BBB disruption. 3000 MW Dextran, sufficiently small to extravasate through disrupted BBBs, but too large to passively cross intact ones, allowed specific visualization of pathological permeability [14, 15]. Confocal imaging was correlated with TSPO-PET signals of the same mice to disentangle the contribution of the BBB disruption, known to be affected in gliomas [16], to the unblocked (i.e. non-specific) TSPO-PET signal.

## Materials and methods

### Study design

This study combines PET imaging, blocking studies, scRadiotracing and intravenous Dextran injection to quantify the impact of BBB disruption on TSPO-PET signals at cellular resolution in experimental glioblastoma. Dynamic [^18^F]GE-180 PET imaging was used in a syngeneic immunocompetent SB28 glioblastoma mouse model. We performed a cross-sectional study design, conducting 60 min TSPO-PET scans after [^18^F]GE-180 application in mice with tumor cell or sham injection at early-stage (week-1) and late-stage (week-3) post inoculation. To independently validate PET imaging findings, a subset of experiments was additionally performed using [^18^F]DPA-714 as a second TSPO radioligand. Prior to scans, a subgroup of animals received blocking by 25,000-fold excessive cold tracer. A subset of mice was afterwards sacrificed for scRadiotracing to quantify cellular tracer uptake, while another was used for confocal microscopy. PET quantification in lesions was performed by calculation of volumes of distribution (VT) using carotid time-activity curves for calculation of image-derived input functions (IDIF). TSPO-PET signals of tumors were extracted using optimized thresholds as described previously [17]. To quantify the magnitude of specific TSPO-PET signals, we performed group-wise comparisons of VTs between blocked and unblocked SB28 and sham mice at early- and late-stage. Animals of the same groups underwent scRadiotracing to pinpoint specificity of cellular [^18^F]GE-180 tracer uptake. BBB disruption in SB28 mice at both time-points was quantified using confocal micoscropy upon intravenous administration of fluorescent Dextran. Ultimately, correlation of confocal and PET imaging of the same animals served to disentangle the impact of the BBB on non-specific TSPO-PET signals in SB28 glioblastomas.

### Animals

All animal experiments were performed in compliance with the National Guidelines for Animal Protection, Germany, with approval of the regional animal committee (Regierung von Oberbayern) and overseen by a veterinarian. All animals were housed in temperature- and humidity-controlled environment with a 12-h light–dark cycle, with free access to food (Ssniff, Soest, Germany) and water. 12-week-old C57BL/6 wildtype (WT) mice were obtained from Charles River Laboratories in Sulzfeld, Germany and acclimated ≥ 1 week. All experiments were performed in female mice. On day 0, mice were inoculated as described previously [9] with 100,000 SB28 GFP(+) cells suspended in Dulbecco´s Modified Eagle Medium (DMEM) (Merck) (SB28 mice, *n* = 76) or saline (sham mice, *n* = 36). Mice were anesthetized for inoculation with intraperitoneal injections of 100 mg/kf ketamine 10% and 10 mg/kf xylazine 2% in 0.9% NaCl, checked daily for tumor-related symptoms (appearance, coordinative deficits, motor symptoms) and sacrificed when stop criteria were reached (not reached in any animal).

### Cell culture

SB28 is a newly developed mouse cell line, not expressing detectable CD40 and representing a weakly immunogenic glioma model [18]. SB28-GFP were cultured in DMEM containing MEM non-essential amino acids (1x), 1% Penicillin-Streptomycin solution (Thermo Fisher Scientific) and 10% fetal bovine serum (Biochrome). Cell cultures were kept in incubators at 37 °C in humidified, 5% CO_2_-conditioned atmosphere and passaged when cell density reached 80% confluence.

### Radiosynthesis

Production of [^18^F]GE-180 and [^18^F]DPA-714 was performed as described previously [17, 19]. For blocking, a 7,15mM solution was created using non-radioactive GE-180 or DPA-714 in Dimethyl sulfoxide (DMSO).

### PET imaging and reconstruction

All small animal PET (µPET) procedures followed an established standardized protocol for acquisition and post-processing [20, 21]. Starting with injection of [^18^F]GE-180 or [^18^F]DPA-714 (21 MBq ± 1.5 MBq) PET data were acquired for 60 min to measure cerebral TSPO expression using a small animal Mediso PET/CT system (Mediso Ltd.) under isoflurane anesthesia (1.5% at time of tracer injection and during imaging; delivery 3.5 L/min). PET reconstruction was performed as described previously [17]. X-ray computed tomography (CT) scans enabled attenuation correction. *N* = 50 mice intravenously recieved cold GE-180 (7,15mM, 25,000-fold excess), while *n* = 62 mice were injected intravenously with contrast medium (0.3 mL, Iomeprol 300 M) 5 min before the CT scan. An overview of animals per group is shown in Supplemental Table 1. The blocking dose was based on previous in vivo blocking experiments [22] and was increased to ensure effective TSPO blockade in tumor-bearing animals with anticipated high TSPO expression. A subset of animals received repeated imaging with and without blocking (1 day apart). Resulting PET images had 212 × 212 × 235 voxels of 0.4 × 0.4 × 0.4 mm^3^. Data were binned to 28 frames, consisting of 6 × 10 s, 6 × 30 s, 6 × 60 s, and 10 × 300 s. Resulting CT images had 972 × 972 × 930 voxels of 0.12 × 0.12 × 0.12 mm^3^.

### PET preprocessing and quantification

All PET analyses were performed using Version 3.4 of PMOD (PMOD Technologies). VT images of the whole mouse were calculated with an IDIF [23] using methodology described by Logan et al. implemented in PMOD [24]. A detailed description can be found in the Supplemental Methods. Non-linear PET coregistration of the brain was performed via CT to brain CT template, with spatial normalization parameters described previously [21]. PET lesion volume of interest (VOI) in SB28 mice was obtained in each animal as described before [17]. In sham mice and contralateral hemispheres, spheres of 1.5 mm radius served for assessment of TSPO-PET signals. Additionally, tumors of mice with blocked and unblocked PET scans at two consecutive days was segmented based on signal intensity of the unblocked scan (*n* = 20 k-mean clusters). VT of blocked and unblocked scans were extracted for all twenty clusters and correlated within groups of *n* = 5 early-stage and *n* = 5 late-stage mice.

### Mouse brain and tumor dissociation

*N* = 60 wildtype C57BL/6 mice with implanted SB28 glioblastomas at early- (*n* = 9) and late-stage (*n* = 29) as well as sham mice (early-stage: *n =* 9, late-stage: *n* = 13) underwent scRadiotracing [12, 25]. Mice were sacrificed by cervical dislocation 75 min after [^18^F]GE-180 administration. *N* = 17 mice received blocking, whereas *n* = 43 did not. See Supplemental Table 2 for an overview of animals per group. Late-stage SB28 tumors were carefully extracted. At early-stage, cubes containing the entirety of tumor tissue were harvested at injection site, as were all contralateral hemispheres and comparably sized tissue samples from sham animals. All samples were dissociated using suitable dissociation kits according to the manufacturer´s instruction as described before [9]. A detailed description is provided in the Supplemental Methods.

### Isolation of tumor and myeloid cells

Tumor and myeloid cell isolation was performed on a total of *n* = 60 samples via Magnetic Cell Sorting (MACS) according to the manufacturer´s instructions as described before [9] and provided in detail in the Supplemental Methods. Afterwards, staining cell pellets with fluoro-chrome-conjugated antibodies recognizing mouse CD11b (Miltenyi Biotec, 130-113-810) enabled further assessment via flow cytometry.

### Calculation of radioactivity per single cell

Radioactivity (in becquerels) of cell pellets were measured in a highly sensitive gamma counter (Hidex) with decay-correction to time of tracer injection. Absolute numbers of tumor and myeloid cells within the pellets were determined via flow cytometry using a MACSQuant10 (Miltenyi Biotec). Ultimately, measured radioactivity was divided by specific cell number, resulting in calculated radioactivity per cell, which was then normalized by injected radioactivity and body weight (i.e., %ID*BW).

### Calculation of radioactivity of extracted tissue samples

A subset of mice were sacrificed after [^18^F]DPA-714 scans with (*n* = 1 week-3 SB28, *n* = 2 sham) and without (*n* = 2 week-3 SB28, *n* = 2 sham) prior blocking via cold DPA-714. Brains were harvested and segmented into tumor, sham injection site and contralateral hemispheres as described above and measured in the gamma counter with decay-correction to time of tracer injection. Normalization was performed by weight of the sample as well as injected radioactivity and body weight.

### Tissue preparation and confocal microscopy

*N* = 5 week-1 and *n* = 5 week-3 SB28 mice were sacrificed by transcardial perfusion 45 min after intravenous injection of 80 µl 3000 MW lysine fixable Dextran (final concentration 20 mg/ml), Texas Red (TR) (Cat No: D3328, Thermo Fischer Scientific, USA) under isoflurane anesthesia for determination of BBB permeability [26, 27]. Three minutes of perfusion with PBS were followed by four minutes with PFA, maintaining constant perfusion pressure of 120 mmHg, as determined ideal for BBB disruption analysis [28].

Brains were post-fixed in 4% PFA at 4 °C for 24 h and subsequently transferred to 0.01% sodium azide PBS at 4 °C to prevent microbial growth. Coronal brain slices (80 μm thick) were prepared using a sliding vibratome (VT1000S, Leica Biosystems, Germany) and stored in 0.01% sodium azide PBS. Two sections from the region of maximal tumor diameter were mounted on glass slides, air-dried for 15 min, and covered with coverslips for imaging. Confocal imaging was performed using a Zeiss LSM 980 confocal microscope (Carl Zeiss Microscopy GmbH, Germany) and image acquisition was carried out using ZEN software (Carl Zeiss Microscopy GmbH, Germany). Whole coronal sections were acquired using a 10× objective in tile scan mode. Images were captured at a resolution of 1024 × 1024 pixels with 5 μm z-steps and bit depth of 16 bits per channel. Two fluorescence channels were acquired sequentially to avoid spectral overlap: GFP (tumor tissue) and TR (vascular tracer). Quantification was performed using the Intensity Sum, calculated by summation of the intensity values of all voxels within the 3D rendered volume and expressed in arbitary units (a.u.).

### Image processing

Image processing and 3D surface reconstruction were performed using Imaris software (version 10.2.0, Oxford Instruments, Switzerland). Image stacks were converted using Imaris File Converter, and voxel dimensions manually set based on acquisition parameters. Surface reconstruction was performed separately for each fluorescence channel using Surface module. Smoothing was enabled with a surface grain size of 0.518 μm for both channels. Quantitative analysis was carried out using the Statistics module, and surface-based measurements including volume, surface area and intensity values were exported as CSV files for downstream analysis.

### Statistics

All statistical analyses were conducted with GraphPad Prism (V10.4.1, GraphPad Software, USA). A p-value < 0.05 was considered significant. PET signals at tumor sites and in contralateral hemispheres were compared between SB28 and sham mice at week-1 and week-3 using one-way ANOVA including a Sidak post-hoc test. For scRadiotracing data, equal standard deviation was not assumed and therefore, one-way ANOVA including a Brown-Forsythe and Welch test was performed to compare radioactivity per cell (tumor cells, TAMs, other cells) in unblocked and blocked conditions at week-1 and week-3. For comparison of histology findings and PET signal as well as PET signals in clusters of blocked and unblocked PET scans, Pearson’s coefficient of correlation was used.

## Results

### Stage-dependent reduction of glioblastoma TSPO-PET signal by blocking with excessive cold ligand

**Fig. 1:**
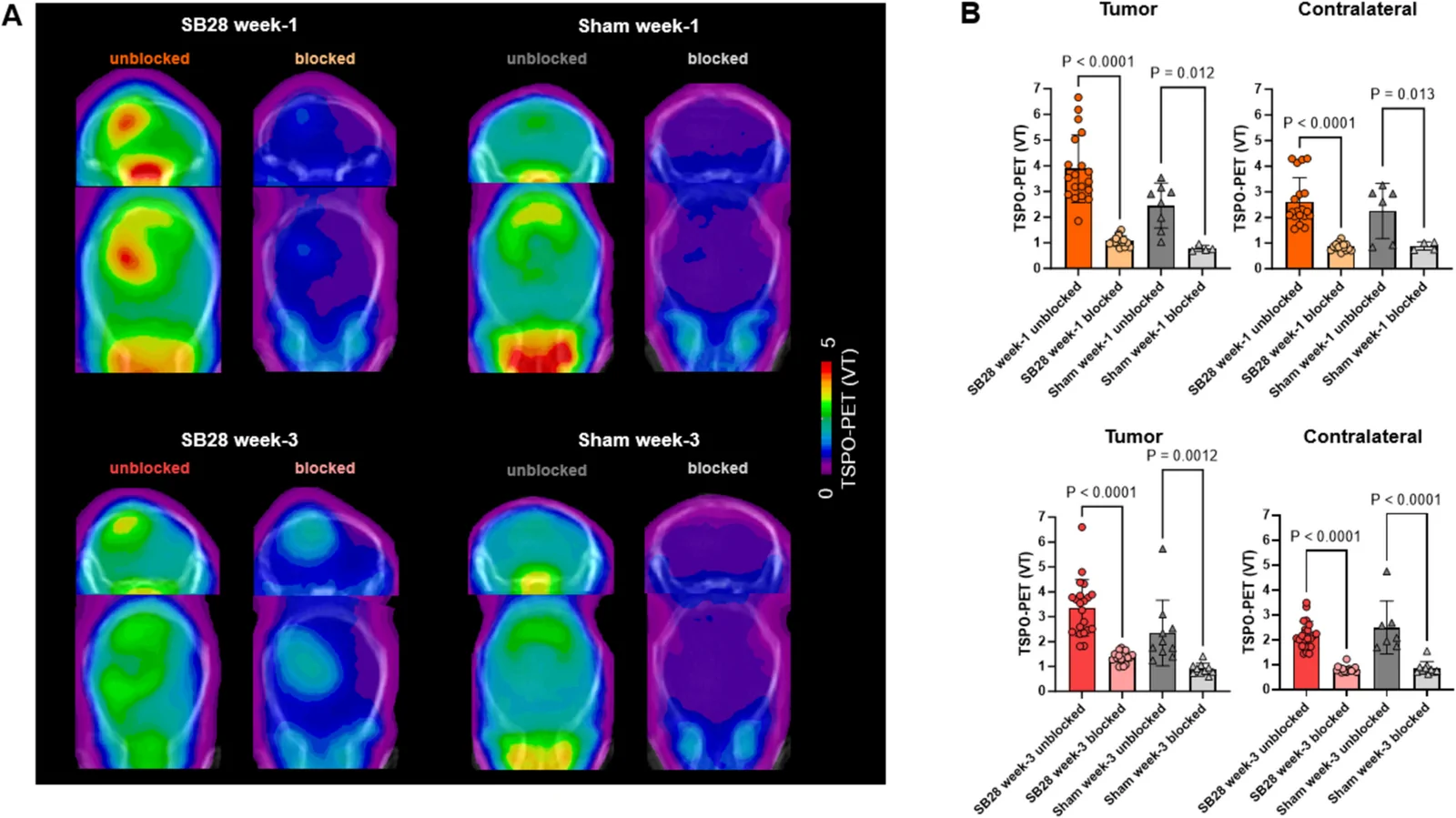
Significant reduction of TSPO-PET signals in early- and late-stage glioblastoma mice upon blocking via cold tracer. (**A**) Group average [^18^F]GE-180 PET images (VT) of SB28 and sham animals before and after blocking with cold tracer. (**B**) Quantification of TSPO-PET signals in early- and late-stage SB28 and sham animals with and without prior blocking with cold tracer

In early-stage SB28 tumors, TSPO-PET signal was reduced by 72% comparing VTs of unblocked (*n* = 19) and blocked (*n* = 15) animals (*p* < 0.0001). Contralateral hemispheres of the same animals showed signal reduction of 65% (*p* < 0.0001). In late-stage SB28 tumors, lower signal reduction (-59%, *p* < 0.0001) between unblocked (*n* = 18) and blocked (*n* = 21) animals was observed, which also applied to contralateral hemispheres (-63%, *p* < 0.0001). In early-stage sham animals, TSPO-PET signal reduction at the inoculation site was 68% (*n* = 8 unblocked vs. *n* = 4 blocked, *p* = 0.012), whereas in late-stage sham animals, signal reduction was 61% (*n* = 10 unblocked vs. *n* = 10 blocked, *p* = 0.0012). In the contralateral hemispheres of early-stage sham animals, signal reduction of 61% (*p* = 0.013) was achieved, in late-stage sham animals signal reduction was 65% (*p* < 0.0001, Fig. 1A, B). Time-activity curves confirmed lower degrees of blocking in week-3 SB28 tumors compared to week-1, contralateral tissue and sham mice (Supplemental Fig. 1). Importantly, the absolute reduction in VT after blocking was higher in tumor regions compared with contralateral and sham tissue (i.e. non-tumor tissue; early-stage: -2.8 vs. -1.7; late-stage: -2.0 vs. -1.5; Supplemental Fig. 2A), consistent with a higher amount of specifically blockable TSPO-associated tracer uptake in tumor regions. To further validate these findings using an independent TSPO radioligand, additional PET imaging with [^18^F]DPA-714 was performed. Prior blocking with cold DPA-714 resulted in similar reductions of the TSPO-PET signal compared with [^18^F]GE-180 (Supplemental Fig. 2B, C). *Ex vivo* gamma counting of extracted tissue upon tracer injection confirmed the in vivo PET findings, demonstrating a 55% reduction in radioactivity following blocking in the tumor, and 28% in non-tumor tissue (Supplemental Fig. 2D).

### Cellular TSPO tracer uptake in SB28 tumors shows strong specificity upon blocking

**Fig. 2:**
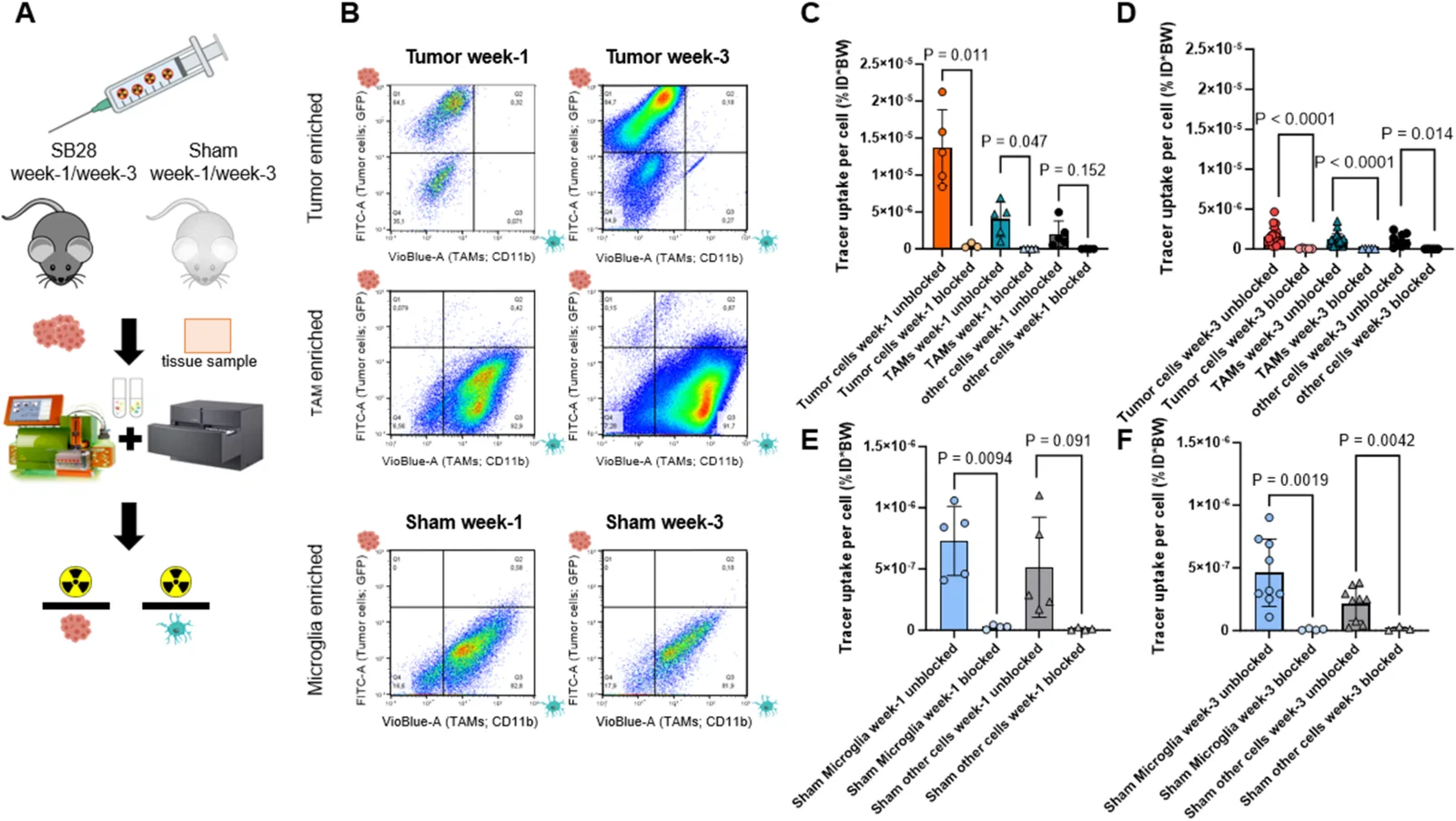
scRadiotracing reveals complete blocking of single-cell tracer uptake upon application of cold tracer. (**A**) Experimental setup of scRadiotracing. Blocking via cold tracer was followed by intravenous application of [^18^F]GE-180. Radioactivity per single cell in blocked and unblocked tumor and sham animals served as the read-out of the experiment. (**B**) Pooled results of flow cytometry analysis of enriched cell pellets (tumor and myeloid cells) at both time-points in tumor and sham animals. (**C**-**F**) Group-wise comparison across all conditions and cell types

Next, we questioned the reduction of single-cell tracer uptake upon blocking with cold ligand using scRadiotracing in enriched pellets of tumor cells, TAMs and remaining cells of the tumor site (Fig. 2A, B). At early-stage, reduction of single-cell radioactivity after blocking was 98% in tumor cells (*n* = 5 unblocked vs. *n* = 3 blocked; *p* = 0.011), 99% in TAMs (*n* = 5 unblocked vs. *n* = 4 blocked; *p* = 0.047) and 99% in the remaining cells (*n* = 5 unblocked vs. *n* = 4 blocked; *p* = 0.152; Fig. 2C). At late-stage, signal reduction was 99% in tumor cells (*n* = 24 unblocked vs. *n* = 5 blocked; *p* < 0.0001), 96% in TAMs (*n* = 24 unblocked vs. *n* = 5 blocked; *p* < 0.0001) and 99% in the remaining cells (*n* = 8 unblocked vs. *n* = 5 blocked; *p* = 0.014; Fig. 2D). Early-stage sham animals showed 99% signal reduction in CD11b(+) cells (*n* = 5 unblocked vs. *n* = 4 blocked; *p* = 0.0094) as well as 99% in the remaining cells (*n* = 5 unblocked vs. *n* = 4 blocked; *p* = 0.091, Fig. 2E). In late-stage sham animals, 98% signal reduction was observed in CD11b(+) cells (*n* = 9 unblocked vs. *n* = 4 blocked; *p* = 0.0019; Fig. 2F). Signal of the remaining cells was reduced by 95% (*n* = 9 unblocked vs. *n* = 3 blocked; *p* = 0.0042; Fig. 2F).

### BBB disruption correlates with residual TSPO-PET signals in SB28 tumors upon blocking

**Fig. 3:**
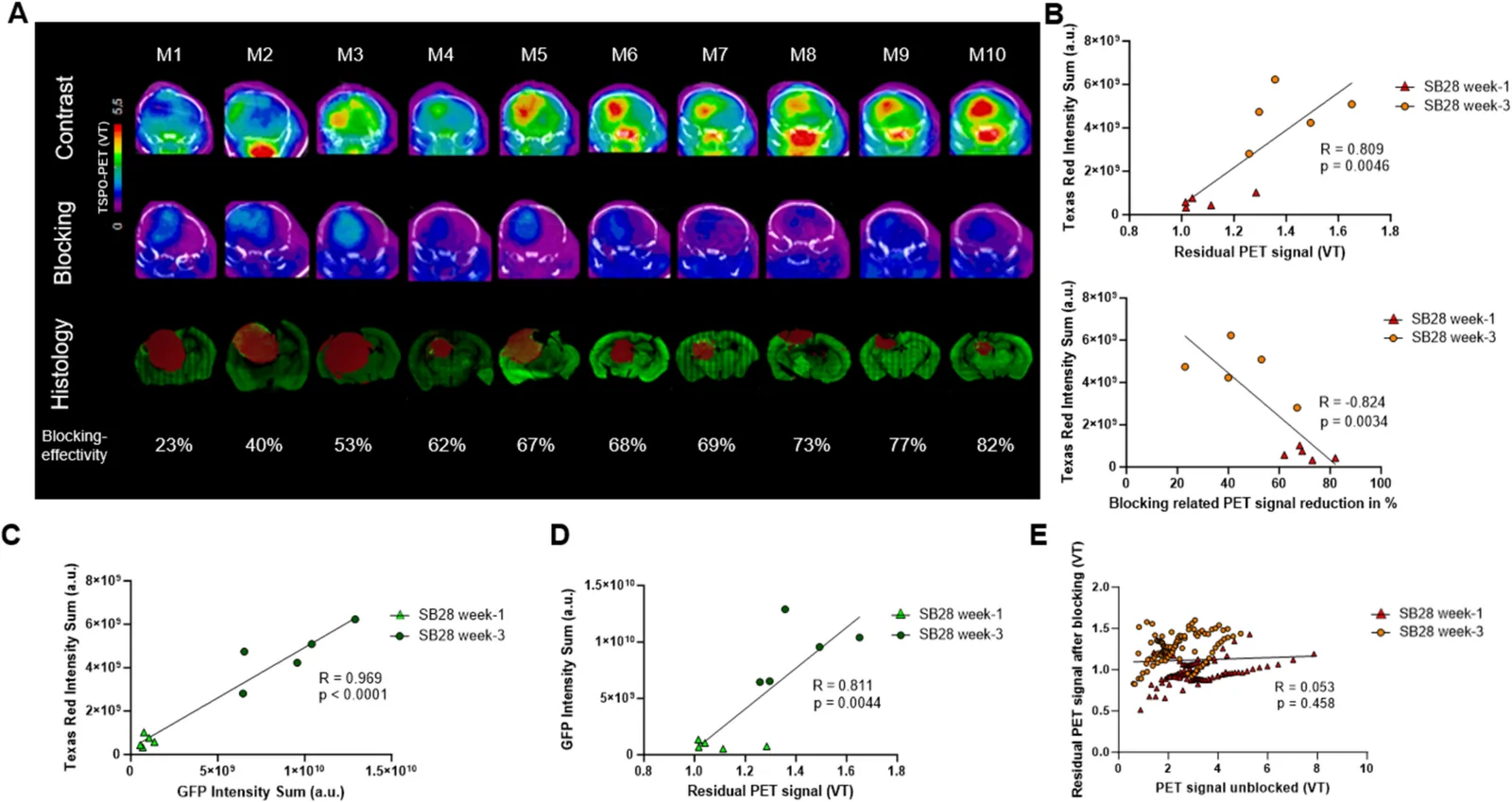
Non-specific PET signals are associated with extent of BBB disruption. (**A**) TSPO-PET images of the same mice (M1–M10 in columns) acquired before (top row) and after (middle row) blocking with cold tracer. Bottom row shows representative confocal overview images from the respective mice after intravenous Dextran application visualizing BBB disruption. Blocking-effectivity refers to the percentage signal reduction in PET between unblocked and blocked conditions. (**B**) Correlation of TR Intensity Sum (read-out for Dextran extravasation) with residual PET signals and PET signal reduction upon blocking. (**C**) Inter-correlation between TR Intensity Sum and GFP Intensity Sum. (**D**) Correlation of GFP Intensity Sum and residual TSPO-PET signals. (**E**) Regional correlation of individual TSPO-PET signals before and after blocking with cold tracer using k-means clustering

To assess BBB disruption in SB28 tumors, confocal microscopy upon intravenous administration of Dextran conjugated with the fluorophore Texas Red was performed (*n* = 5 week-1, *n* = 5 week-3 SB28 tumors; Fig. 3A). TR Intensity Sum was 6.3 ± 2.7*10^8^ a.u. at early-stage and increased to 4.6 ± 1.3*10^9^ a.u. towards late-stage tumors (*p* = 0.0001), indicating increased Dextran extravasation following tumor progression. Importantly, there was a significant correlation of Dextran intensity with residual TSPO-PET signal (*R* = 0.809, *p* = 0.0046) as well as PET signal reduction after blocking with cold ligand (*R*=-0.824, *p* = 0.0034; Fig. 3B). Furthermore the inter-correlation between TR Intensity Sum and GFP Intensity Sum (surrogate of tumor volume) indicated higher Dextran extravasation in larger tumors (*R* = 0.969, *p* < 0.0001; Fig. 3C). In line, residual PET signal after blocking correlated with GFP Intensity Sum (*R* = 0.811, *p* = 0.0044; Fig. 3D), pointing towards more unspecific signal in large tumors. In contralateral hemispheres, we found substantially less Dextran intensity compared to SB28 tumors at early- and late-stage (Supplemental Fig. 2E). No significant correlation between TR Intensity Sum and residual TSPO-PET signal (*R* = 0.553, *p* = 0.098) or PET signal reduction after blocking (*R*=-0.155, *p* = 0.670) in contralateral hemispheres was observed (Supplemental Fig. 2F, G).

### Allocation of TSPO-PET signals to cellular and extracellular compartments in dependence of BBB disruption

**Fig. 4:**
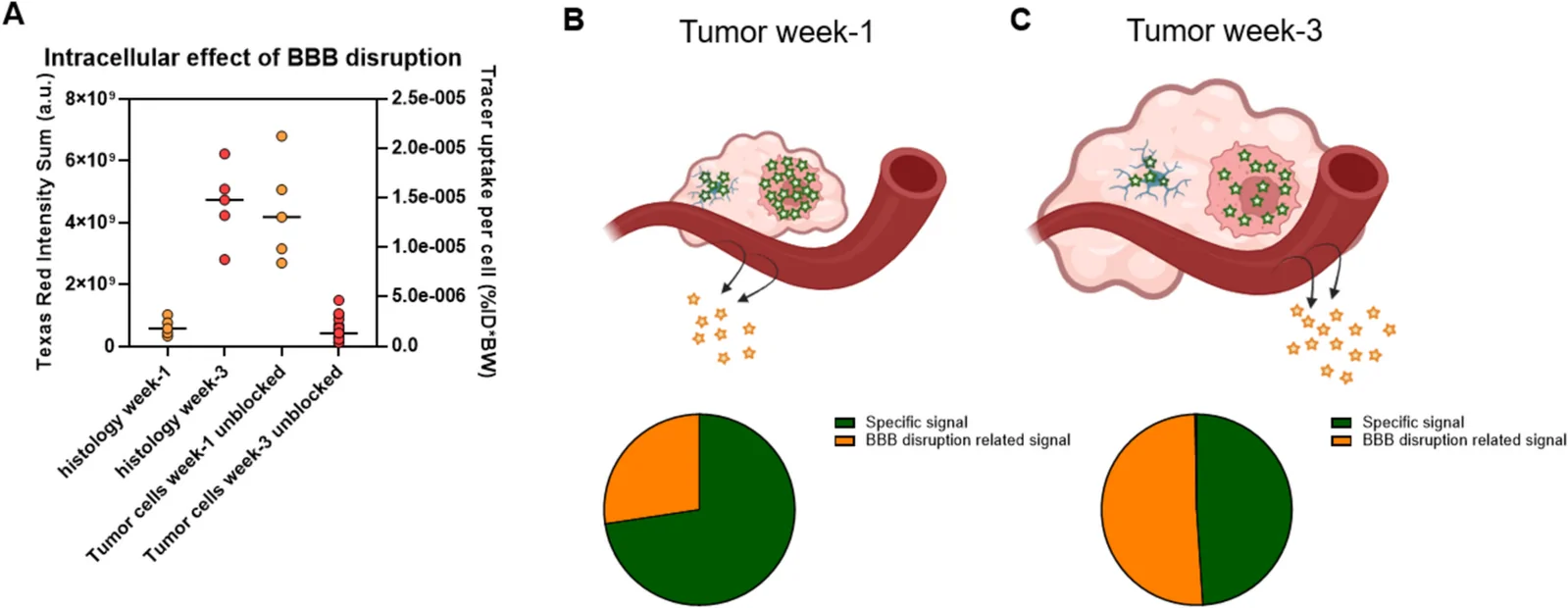
Allocation model of TSPO-PET signals in SB28 glioblastoma. (**A**) Illustration of the magnitude of TR Intensity Sum and single-cell TSPO tracer uptake in early- and late-stage SB28 tumors. (**B**, **C**) In early-stage SB28 tumors, 72% of TSPO-PET signal derives from specific binding of tracer to the cellular compartment. In late-stage tumors, 59% of signal is due to specific binding. The remaining signal is explained by tracer extravasation through disrupted BBBs. Upper row: Schematic illustration of GE-180 allocation to cellular and extracellular compartments. Specific signal is illustrated as green stars, unspecific signal as orange stars respectively. Lower-row: Pie chart illustrating the distribution of specific (green) and unspecific (orange) signal at both time-points

Ultimately, we combined the acquired experimental data to determine allocation of TSPO tracer uptake in different conditions (early- and late-stage SB28 and sham) to cellular and extracellular compartments of SB28 glioblastoma. Since cellular signal in all conditions was blocked by ≥ 95% in TAMs, tumor cells and remaining cells of the tumor site, all non-specific tracer uptake was attributed to the extracellular compartment; its magnitude strongly correlating with the extent of Dextran extravasation indicative of BBB disruption (Fig. 3B). The remaining question consisted in potential impact of BBB disruption on specific cellular PET signals. Due to divergent trajectories of cellular TSPO tracer uptake (decrease) and Dextran extravasation (increase) during development of SB28 tumors (Fig. 4A), we anticipated low likelyhood for BBB-driven increases of cellular TSPO tracer uptake. Since direct correlation of Dextran with scRadiotracing was technically not possible, we leveraged regional tumor heterogeneity to pinpoint if residual PET signals after blocking as a surrogate of regional BBB disruption affect specific PET signals (Δunblocked-blocked) in tumor clusters (Fig. 3E). There was no correlation between regional residual PET signals and specific PET signals (*R* = 0.052, *p* = 0.458). Thus, in summary, TSPO-PET signals in SB28 tumors consist of a specific cellular component driven by target expression of tumor cells and TAMs and an extracellular component driven by tracer leakage through an open BBB (Fig. 4B, C).

## Discussion

This is the first study that exploits combining PET imaging, blocking experiments, cell sorting after radiotracer injection and intravenous Dextran injection to decipher the impact of BBB disruption on cellular and extracellular radiotracer uptake. For this purpose, we used [^18^F]GE-180 as a TSPO-PET tracer with supposedly high dependency on BBB integrity. By blocking with cold tracer, we show that 72% of TSPO-PET signals in early-stage SB28 tumors result from specific binding, whereas increasing impact of BBB disruption leads to higher shares of non-specific signal in late-stage tumors. Using scRadiotracing, we managed to fully allocate specific TSPO-PET signals to the cellular compartment of tumor cells, TAMs and remaining cells of the glioblastoma TME. Confocal micoscropy upon intravenous administration of Dextran deciphered that non-specific TSPO-PET signal was highly dependent on BBB disruption, whereas regional analysis of tumor clusters indicated that specific cellular tracer uptake was not driven by tracer leakage through an open BBB.

A major strength of our study relates to the joint use of PET imaging, cell sorting after tracer injection and assessment of BBB disruption, allowing us to resolve a longlasting debate on the impact of BBB disruption on [^18^F]GE-180 PET signals, a TSPO radiotracer known for low brain penetration in physiological conditions [29]. Our data show that specific TSPO-PET signal can exceed 70%, but that the variable contribution of BBB disruption needs to be considered during interpretation of TSPO-PET images in glioblastoma. These findings help reconcile the ongoing discussion regarding the interpretation of [^18^F]GE-180 uptake in brain tumors. While limited physiological brain penetration has raised concerns that [^18^F]GE-180 signals may predominantly reflect BBB disruption rather than specific TSPO binding [10, 11], clinical and preclinical studies have consistently demonstrated robust tracer accumulation in TSPO-expressing gliomas [8, 9]. Our data indicate that both views capture distinct aspects of TSPO-PET biology: specific cellular binding accounts for the majority of the signal in early-stage tumors, whereas BBB disruption contributes an additional extracellular component during tumor progression. Importantly, cellular TSPO-PET signals were entirely specific, which is a major novel finding introduced by combined application of scRadiotracing and blocking with cold tracer. Previous studies have demonstrated TSPO expression in both TAMs and glioblastoma cells, with recent evidence suggesting particularly high TSPO levels in mesenchymal glioblastomas [6, 8]. The present findings extend these observations by directly demonstrating highly specific radiotracer binding at the cellular level in both tumor cells and TAMs as well as remaining cells within the glioblastoma microenvironment.

The second important topic frequently raised was related to the potential impact of BBB disruption on specific PET signals, i.e. if increased tracer leakage through an open BBB also enhances specific cellular tracer binding. [^18^F]GE-180 poses an ideal tool to answer this question, since the proposed impact should be particularly strong for molecules with limited BBB permeability. Here, we clearly observed no association between the degree of BBB disruption in heterogeneous tumor areas and specific PET signals in the same region, thus arguing against BBB permeability as a major driver of specific PET signals. Importantly, while Dextran extravasation provides a valuable readout of BBB disruption and represents a major strength of our approach, its extent reflects both vascular permeability and the size of the vascular surface area [30, 31]. The strong correlation between Dextran intensity and tumor size observed in our data suggests that increased tracer extravasation in larger tumors may ideed reflect both enhanced permeability and increased vascular density following angiogenesis and tumor growth, which cannot be disentangled based on the present measurements. A regional correlation of tracer extravasation with vascular density could provide further insight into the relative contribution of both factors and the underlying vascular changes. Idenpendent of these specific considerations, our findings are relevant beyond TSPO-PET imaging. BBB disruption represents a major challenge for the interpretation of molecular imaging and therapeutic delivery in glioblastoma [32], as vascular permeability varies substantially across tumor regions [33] and throughout disease progression [27, 34, 35]. Concurrently, the methodological approach presented in this study may likewise be used to study specificity and BBB dependency of other PET tracers used for glioma imaging. Furthermore, the applied experimental setup will also facilitate to stratify cellular and extracellular sources of PET tracer signals in other disease conditions with BBB affection [16].

A counterintuitive finding was the lower overall PET signal observed in late-stage tumors compared with early-stage, despite more pronounced influence of BBB disruption and increased abundance of tumor and myeloid cells during tumor progression. Importantly, this finding was independent of the quantification approach, as both VT and standardized uptake values (SUV) showed a comparable early-to-late-stage decline in our previous study [17]. Interestingly, a decline in cellular tracer uptake from early- to late-stage tumors is also evident in our scRadiotracing data. Phenotypic changes of tumor and myeloid cells are well known to occur during tumor progression [36, 37] and may also affect TSPO expression and cellular tracer uptake, potentially counterbalancing the increased abundance of tumor and myeloid cells and the more pronounced impact of BBB disruption at later stages. In addition, progressive tumor necrosis [38] may reduce the fraction of viable, TSPO-expressing tissue and thereby contribute to lower cellular tracer retention and overall TSPO-PET signals. Progressive vascular remodeling [39] may further alter local perfusion and tracer delivery, resulting in heterogeneous tracer distribution within late-stage tumors. Thus, while BBB disruption may increase extracellular tracer availability, PET signals reflect a complex interplay of multiple biological and vascular factors during tumor progression.

In non-tumor conditions (i.e. contralateral and sham), we observed similar degrees of relative blocking compared to early-stage SB28 tumors. However, analysis of absolute VT changes after blocking revealed substantially higher reductions in tumor tissue compared with non-tumor regions, indicating a greater amount of specifically blockable TSPO-associated signal in tumor tissue. In contrast, the similar relative reductions observed in non-tumor tissue are likely influenced by higher contributions of background signals relative to the total signal in regions without elevated TSPO expression [40]. Furthermore, the absence of relevant Dextran extravasation support the interpretation that residual signal in non-tumor tissue is not primarily driven by BBB disruption. Spillover and partial-volume effects represent important considerations in small-animal PET imaging [41], particularly when assessing normal-appearing tissue adjacent to the tumor lesion. However, our ex vivo gamma counting approach supports that the observed contralateral blocking effect reflects local tracer binding to e.g. myeloid cells within the contralateral hemisphere [42] rather than mere spillover from tumor or other adjacent tissue.

[^18^F]GE-180 was selected as the primary tracer for this study, as its limited brain penetration and reported dependence on BBB integrity make it particularly suitable to investigate the impact of BBB alterations on TSPO-PET signal distributions in our experimental model. Nevertheless, to ensure that our findings were not dependent on tracer-specific properties, we additionally validated our results using the independent TSPO radioligand [^18^F]DPA-714 [43], which furthermore represents an important strength of the study. Comparable blocking effects were observed with [^18^F]DPA-714, suggesting that the observed separation of cellular and BBB-dependent signal components is not restricted to a single TSPO radioligand but likely reflects a general feature of TSPO-PET imaging in glioblastoma.

A limitation is the exclusive use of female mice. Although the present work was not designed to investigate sex-dependent effects but rather to establish a methodological framework for assessing the contribution of BBB integrity to TSPO-PET signals in brain, previous TSPO-PET studies have reported sex-related differences in tracer uptake and TSPO expression [44, 45]. Therefore, future studies including both sexes will be important to determine the generalizability of the present findings. Another limitation is that this study was performed using a single glioblastoma cell line. Given the biological heterogeneity of glioblastoma [46], the methodology established in this study provides a framework that can be applied to additional glioblastoma models to further characterize the impact of tumor-specific properties and BBB alterations on tracer uptake.

## Conclusion

Overall, we observed variable degrees of non-specific TSPO-PET signals in mice with SB28 glioblastoma, which were fully allocated to the extracellular compartment and driven by BBB disruption. Contrary, cellular TSPO-PET signals were entirely specific and not influenced by the extent of tracer extravasation across the disrupted BBB. 

## Supplementary Information

Below is the link to the electronic supplementary material.

Supplementary Material 1

## Data Availability

The datasets generated during and/or analysed during the current study are available from the corresponding author on reasonable request.
