# Supplementary material for: Allocation of TSPO-PET signals to cellular and extracellular compartments in dependence of blood-brain barrier disruption in experimental glioblastoma
Source: Eur J Nucl Med Mol Imaging. 2026 Sep 24;53(13):8675–85. doi: 10.1007/s00259-026-08187-5 (PMC13727962; doi:10.1007/s00259-026-08187-5)
Supplement: Supplementary file 1 [file 259_2026_8187_MOESM1_ESM.docx]

**SUPPLEMENTAL DATA**

**Supplemental Tables**

|  | blocked | unblocked | Total |
| --- | --- | --- | --- |
| Sham week-1 | n=4 | n=8 | n=12 |
| Sham week-3 | n=10 | n=10 | n=20 |
| SB28 week-1 | n=15 | n=19 | n=34 |
| SB28 week-3 | n=18 | n=21 | n=39 |
| Total | n=47 | n=58 | n=105 |

**Supplemental Table 1**: Number of animals scanned with [^18^F]GE-180.

|  | blocked | unblocked | Total |
| --- | --- | --- | --- |
| Sham week-1 | n=4 | n=5 | n=9 |
| Sham week-3 | n=4 | n=9 | n=13 |
| SB28 week-1 | n=4 | n=5 | n=9 |
| SB28 week-3 | n=5 | n=24 | n=29 |
| Total | n=17 | n=43 | n=60 |

**Supplemental Table 2**: Number of animals undergoing scRadiotracing after [^18^F]GE-180 PET scan.

**Supplemental Methods**

**PET processing and quantification**

For calculation of VT images of the whole mouse, blood input curves were obtained from standardized bilateral VOIs placed in both carotid arteries (*23*). Maximum error of 10% and VT threshold of 0 were selected for modeling of full dynamic imaging data with a one-tissue compartment model. Time of the linear fit was set flexible (t*) and uptake rate constant K1 and dissociation rate constant K2 were obtained for lesions.

**Mouse brain and tumor dissociation**

After sacrification of mice, whole brains were extracted and transferred to cold Dulbecco’s phosphate-buffered saline (D-PBS). The SB28 tumors, contralateral hemispheres and tissue samples of sham animals were cut into small pieces and dissociated using gentleMACS Octo Dissociator with Heaters (Miltenyi Biotec, 130-096-427) and their respective, apt dissociation kits. Adult brain dissociation kit, mouse and rat (Miltenyi Biotec, 130-107-677) was used on sham brains and contralateral hemispheres of SB28, Tumor dissociation kit (Miltenyi Biotec, 130-096-730) on tumor tissue. The dissociated cell suspension was carefully applied to 70-μm cell strainer (Miltenyi Biotec, 130-110-916), prewet with D-PBS. After centrifugation at 4°C and 300*g*  for 10 min (acceleration and deceleration at 5), the resulting cell pellet was resuspended in cold D-PBS and debris removal solution. Through gentle overlay of cold D-PBS on top of the cell suspension and centrifugation at 4°C and 3000*g* for 10 min (acceleration at 9, deceleration at 5) three phases emerged. The two top phases were removed entirely, the remaining cell pellets collected and resuspended with 1 ml of cold red blood cell removal solution, followed by 10-min incubation. Afterwards, cell pellets were collected for further applications.

**Isolation of tumor and myeloid cells**

For isolation of tumor cells, Tumor cell isolation kit, mouse (Miltenyi Biotec, 130-110-187), was used according to the manufacturer’s instructions. Previously prepared cell pellets were resuspended in 80 μl of D-PBS–0.5% bovine serum albumin (BSA) buffer per 10^7^ total cells including red blood cells. 20µl of depletion cocktail (nontumor cells) was added per 10^7^ total cells, the suspension then incubated at 4°C in the dark for 15 min. Afterwards, D-PBS–0.5% BSA buffer was added for a total volume of 500 μl before magnetic separation.
For TAMs, the prepared cell pellets were resuspended in 90 µl of D-PBS–0.5% BSA buffer and 10 µl of CD11b MicroBeads, human and mouse (Miltenyi Biotec, 130-049-601) per 10^7^ total cells. After incubation for 15 min in the dark at 4°C, cells were washed by adding 1 ml of buffer per 10^7^ cells and centrifuged at 300*g* for 10 min (acceleration and deceleration at 5). The cell pellets were then resuspended in 500 μl of D-PBS–0.5% BSA
For both cell types, prewet LS columns (Miltenyi Biotec, 130-042-401) were placed at QuadroMACS Separator (Miltenyi Biotec, 130-090-976). The cell suspensions were applied onto the column, which were then washed multiple times with D-PBS–0.5% BSA buffer.

For tumor cells, the flow-through containing the unlabeled cells was collected as the tumor cell–enriched fractions The columns were removed from the magnetic field, and the nontumor cells were flashed out using 3 ml of D-PBS–0.5% BSA buffer.

For TAMs, the flow-through contained the nonTAM cells, while the TAMs were magnetically labeled. Therefore, they were washed out with 5ml of D-PBS–0.5% BSA buffer after being removed from the magnetic field.

**Supplemental Figures**

**Supplemental Figure 1: Time-activity curves of the tumor and contralateral site in early- and late-stage SB28 glioblastoma**

(A) Time-activity curves of early- and late-stage SB28 glioblastoma mice within the tumor site (left) and contralateral hemisphere (right) before and after blocking with cold tracer.

(B) Time-activity curves of early- and late-stage sham mice within the inoculation site (left) and contralateral hemisphere (right) before and after blocking with cold tracer.

(C) Time-activity curves of unblocked early- and late-stage SB28 glioblastoma mice within the tumor site and contralateral hemisphere.

(D) Time-activity curves of unblocked late-stage SB28 glioblastoma mice within the tumor site, contralateral hemisphere, and tumor site after exclusion of animals with visually marked necrosis on TSPO-PET.

**Supplemental Figure 2: Complementary analyses of TSPO-PET blocking effects and BBB permeability**

(A) TSPO-PET signals in SB28 tumors and corresponding non-tumor tissue (sham injection site and contralateral hemispheres) of blocked and unblocked animals at early- and late-stage. Numbers above brackets indicate absolute differences in VT between blocked and unblocked groups.

(B) Group average [^18^F]DPA-714 PET images (VT) of late-stage SB28 and sham animals before and after blocking with cold tracer.

(C) Relative reduction of TSPO-PET signals following blocking in tumor and sham animals comparing [^18^F]GE-180 and [^18^F]DPA-714.

(D) *Ex vivo* gamma counter measurements of dissected SB28 tumors and corresponding non-tumor tissue (sham injection site and contralateral hemispheres) after [^18^F]DPA-714 administration.

(E) Quantification of TR Intensity Sum in tumor and contralateral hemispheres of early- and late-stage SB28 mice.

(F, G) Correlation of TR Intensity Sum with residual PET signals and PET signal reduction upon blocking in contralateral hemispheres.
